# Evidence that an *APOE *ε4 'double whammy' increases risk for Alzheimer's disease

**DOI:** 10.1186/1741-7015-10-36

**Published:** 2012-04-13

**Authors:** Ina Caesar, Sam Gandy

**Affiliations:** 1 Department of Neurology, Mount Sinai School of Medicine, One Gustave L. Levy Place, New York NY 10029; 2 Department of Psychiatry, Mount Sinai School of Medicine, One Gustave L. Levy Place, New York NY 10029; 3 Alzheimer's Disease Research Center, Mount Sinai School of Medicine, One Gustave L. Levy Place, New York NY 10029; 4 James J Peters VA Medical Center, 130 Kingsbridge Road, New York NY 10468, USA

**Keywords:** Alzheimer's disease, epilepsy, apolipoprotein, cerebral amyloidosis

## Abstract

Temporal lobe epilepsy (TLE) is associated with some of the same neuropathological features as those reported for early stages of typical Alzheimer's disease (AD). The *APOE ε4 *allele is associated with a gene-dose-dependent increase in AD risk and in the severity of amyloid-β (Aβ) pathology. In a study published in the current *BMC Medicine*, Sue Griffin and colleagues studied markers of brain resilience in the amputated temporal lobes of TLE patients. They discovered compelling evidence that the *APOE ε3 *isoform in TLE patients is apparently more neuroprotective from Aβ toxicity than is the *APOE ε4 *isoform, as shown by the reduced levels of neuronal damage, glial activation, and expression of IL-1α in the *APOE ε3/ε3 *brains. This result points to a new property of *APOE *isoforms: not only are *APOE ε4 *alleles associated with increased brain amyloid plaque burden, but these alleles are also apparently inferior to *APOE ε3 *alleles in conveying resistance to Aβ neurotoxicity. This 'double whammy' result opens up a new direction for studies aimed at elucidating the relevant neurobiological activities of *APOE *isoforms in the pathogenesis of AD.

Please see related article: http://www.biomedcentral.com/1741-7015/10/35

## Background

Alzheimer's disease (AD) is a progressive neurological disease and is the most common form of dementia. The prevalence of AD is aging-related: 10% of people over 65 years old and 50% of people over 85 years old are affected. The disease course involves unrelenting decortication, with affected people succumbing in a persistent vegetative state after an approximate 10-year course of illness. AD is characterized by the accumulation of extracellular amyloid plaques and intraneuronal neurofibrillary tangles as well as profound neuronal loss. This neuronal loss correlates better with clinical cognitive status at the time of death than does the burden of either pathology; therefore, an understanding of the molecular mechanisms of neurotoxicity (and 'neuroresilience') in AD is of paramount interest.

Apolipoprotein E ε4 (*APOE ε4*, gene; apoE4, protein) is the major identified genetic risk factor for common sporadic forms of AD, and understanding the relevant neurobiological activities of *APOE *isoforms has presented a major challenge over the past 20 years. Temporal lobe epilepsy (TLE) is associated with some of the same neuropathological features as those reported for early stages of typical AD [1]. The changes in neuropathology reported for TLE include accumulation of amyloid-β (Aβ) as senile plaques and increased microglial activation compared to healthy controls. The *APOE ε4 *allele in TLE is associated with a gene-dose-dependent increase in plaque density when compared with brains of *APOE ε3/ε3 *genotype [2]. An important distinction is that the TLE brain is assumed not to be as generally compromised as is the AD brain. Therefore, the plaque-loaded TLE brain presents an unusual opportunity to study focal parenchymal cerebral amyloidosis situated in what is believed to be an otherwise healthy brain.

**Figure 1:**
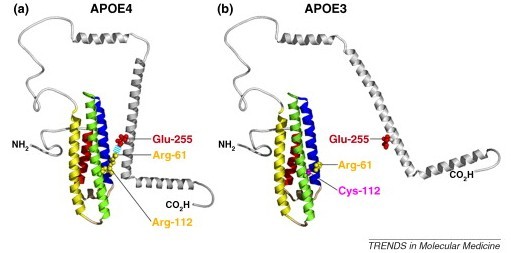
**Structure of apoE3 and apoE4**. The polymorphism at residue 112 (which can be either arginine in apoE4 or cysteine in apoE3) is believed to underlie the differential antioxidant activities of apoE3 and apoE4 (adapted from [5], with permission).

In a research article published in the current issue of *BMC Medicine*, Sue Griffin and colleagues have investigated the brains of TLE patients with *APOE ε3 *and *APOE ε4 *alleles. By focusing on TLE, Griffin avoided the confound of the possible underlying vulnerability to neurotoxicity that might characterize the AD brain. Further, by studying younger subjects than those generally studied in typical AD, the researchers also avoided the possibility of aging-related confounds. This research uncovered compelling evidence that, after correcting for the differences in plaque density, the apoE3 isoform in TLE patients is apparently more neuroprotective from Aβ toxicity than is the apoE4 isoform. This conclusion is supported by the reduced levels of neuronal damage, glial activation, and interleukin-1α (IL-1α) in the *APOE ε3/ε3 *brains [3] and is consistent with the predictions of Miyata and Smith, who provided evidence for the differential antioxidant activities of apoE3 vs apoE4 proteins [4]. The differential antioxidant activities of apoE isoforms are due to the Cys- and Arg- substitutions at the polymorphic site [5], which are illustrated in Figure 1.

### Tissue levels of IL-1α are dependent on *APOE *isoform

IL-1α is one of the best characterized inflammatory cytokines, and, in earlier studies, Griffin and colleagues have previously established that elevation of brain IL-1α is an important feature of AD. The elevation of IL-1α in the TLE cases was apparently related to an increase in the number of activated microglia. Neurons in the *APOE ε3/ε3 *brains had twice as many associated microglia per neuron when compared with the respective data from *APOE ε4/ε4 *brains. The increased numbers of microglia per neuron in *APOE ε3/*ε3 TLE patients were associated with an increased level of IL-1α mRNA and protein. The IL-1α *mRNA *levels were five-fold higher in the TLE patients compared to healthy controls, but those levels were independent of *APOE *isoform. IL-1α protein levels were four-fold higher in *APOE ε3/ε3 *patients than that in *APOE ε4/ε4 *patients. The elevated IL-1α protein levels in the TLE patients apparently resulted in increased amyloid precursor protein (APP) and apoE protein expression as a function of the *APOE *isoform. This supports the idea that microglia activation and elevated IL-1α expression may contribute to the accumulation of APP and apoE, two key proteins known to be important for increasing the risk of AD pathology (see [6], for review), and that this will primarily take place in the *APOE ε4/ε4 *brain.

### Neuronal damage and size are associated with *APOE *isoform

There were no differences in the numbers of neurons in cortical layers III-VI of three specific areas of the temporal lobe in TLE patients depending on *APOE *isoform in the Griffin study. The DNA damage in neurons was also independent of the *APOE *genotype. However, the level of DNA damage *per neuron *was greater in patients with *APOE ε4/ε4 *than in those with the *APOE ε3/ε3 *genotype. Further, the average size of neurons in patients with *APOE ε4/ε4 *genotype was unexpectedly smaller than in patients with *APOE ε3/ε3 *genotype. Neurons from TLE patients with the *APOE ε3/ε3 *genotype were larger in terms of the size of both the cytoplasm as well as the size of their nuclei, and the *APOE ε3/ε3 *neurons appeared to have a more normal morphology than did neurons from TLE patients with the *APOE ε4/ε4 *genotype. Due to these findings, Griffin *et al*. propose that neurons from individuals with *APOE ε3/ε3 *isoform are better protected from the damaging hyperexcitability associated with epilepsy than were the neurons from TLE patients with *APOE ε4/ε4 *isoform. They also noted that this genetic variation of neuronal sparing may result from typical acute phase responses of neurons that result in alternate levels of IL-1α, APP, and apoE expression. These same molecules would be predicted to protect against DNA fragmentation in TLE patients carrying the *APOE ε3 *allele. The basic science mechanisms linking *APOE ε4 *with a reduction in various biomarkers of resilience may, at least in part, underlie the clinical association of the *APOE ε4 *allele with one or more of the negative outcome factors when human TLE is linked to *APOE ε4*, such as increased tendency toward earlier age at onset of TLE and/or bilaterality of hippocampal damage, although not all of these negative TLE/*APOE ε4 *associations have been independently confirmed [7-19]. IL-1α is also genetically linked to the risk for developing TLE in at least two independent studies [20].

### Density of Aβ plaques in TLE patients was associated with *APOE *isoform

Senile plaques are reported in about 10% of all TLE cases and are evident at a younger age than that typically associated with senile plaques in AD [1]. Griffin *et al*. describes the occurrence of Aβ/apoE immunoreactive senile plaques in TLE patients as a function of age, in a distribution similar to that noted in temporal lobes of early stages of typical AD patients. Aβ/apoE immunoreactive senile plaques were even found in a 10-year-old TLE patient, and senile plaques at such early ages suggests that the neuropathology in TLE patients is not likely to be attributable to the coincidental presence of AD. Due to the limited number of patients with the *APOE ε4/ε4 *genotype, this study does not reveal whether a specific *APOE *genotype is associated with either a higher probability of having senile plaques in TLE or with a shift in the age of onset for senile plaque formation. Gouras *et al*. [2] have previously written in this topic, reporting that *APOE ε4 *alleles were associated with onset of plaque pathology below the age of 50 years. The role of amyloid plaques in the clinical phenomena linked to TLE and *APOE ε4 *[7-19] have not been systematically studied, although the recent advent of amyloid plaque imaging PET scanning now enables the investigation required to answer this question.

## Conclusions

Griffin *et al*. show for the first time that the *APOE ε3/ε3 *genotype is apparently more neuroprotective than the *APOE ε4/ε4 *genotype, as judged by neuronal damage, glial activation, and brain IL-1α level in TLE patients. These findings are consistent with the idea that neurons in TLE patients carrying the *APOE ε4 *allele are less resistant to the damaging hyperexcitability associated with epilepsy and, therefore, these neurons are more prone to development of DNA damage. In the setting of aging and AD, however, the lower neuroprotection activity of apoE4 may underlie the increased risk for AD in patients carrying the *APOE ε4 *allele. This finding opens up new avenues for research aimed at elucidating the molecular basis for apoE3-mediated neuroprotection. ApoE3-mimetic drugs are highly sought as potential AD therapeutics as an example of personalized medicine based on individual genetic risk. Recent evidence suggests that bexarotene, a retinoic acid RXR ligand, may be a breakthrough lead in this quest [21]: the drug appears to act through apoE to cause rapid (i.e. within 72 hours) clearance of amyloid plaques in a mouse model of AD. The perfection of apoE-mimetic drugs [22-31] for AD may well have a beneficial side effect of yielding compounds that might be also helpful for mitigating temporal lobe and hippocampal damage in patients with TLE.

## Competing interests

Dr Caesar has no competing interests to declare. Dr Gandy has received honoraria or grants from Pfizer, J&J, Amicus, Diagenic, and Baxter.

## Authors' contributions

Drs Caesar and Gandy each contributed drafts of portions of the original text. Dr Gandy edited the final text. Both authors read and approved the final manuscript.

## Authors' information

Drs Caesar and Gandy are at the Departments of Neurology and Psychiatry and Alzheimer's Disease Research Center, Mount Sinai School of Medicine, and James J Peters VA Medical Center, New York, NY 10029, USA.

## Pre-publication history

The pre-publication history for this paper can be accessed here:

http://www.biomedcentral.com/1741-7015/10/36/prepub

## References

[B1] Mackenzie IR Miller LA Senile plaques in temporal lobe epilepsy Acta Neuropathol 1994 87 504 510 10.1007/BF00294177 8059603

[B2] Gouras GK Relkin NR Sweeney D Munoz DG Mackenzie IR Gandy S Increased apolipoprotein E epsilon 4 in epilepsy with senile plaques Ann Neurol 1997 41 402 404 10.1002/ana.410410317 9066363

[B3] Aboud O Mrak RE Boop FW Griffin ST Apolipoprotein epsilon 3 alleles are associated with indicators of neuronal resilience BMC Med, this issue 10.1186/1741-7015-10-35 PMC3352297 22502727

[B4] Miyata M Smith JD Apolipoprotein E allele-specific antioxidant activity and effects on cytotoxicity by oxidative insults and beta-amyloid peptides Nat Genet 1996 14 55 61 10.1038/ng0996-55 8782820

[B5] Huang Y Abeta-independent roles of apolipoprotein E4 in the pathogenesis of Alzheimer's disease Trends Mol Med 2010 16 287 294 10.1016/j.molmed.2010.04.004 20537952

[B6] Gandy S The role of cerebral amyloid beta accumulation in common forms of Alzheimer's disease J Clin Invest 2005 115 1121 1129 15864339 10.1172/JCI25100 PMC1087184

[B7] Fu YH Lv RJ Jin LR Lu Q Shao XQ He JS Wu LW Zhang LS Hu HG Association of apolipoprotein E polymorphisms with temporal lobe epilepsy in a Chinese Han population Epilepsy Res 2010 91 253 259 10.1016/j.eplepsyres.2010.07.020 20810250

[B8] Busch RM Floden D Lineweaver TT Chapin JS Unnwongse K Wehner T Diaz-Arrastia R Najm IM Effect of apolipoprotein ε4 allele on hippocampal and brain volume in intractable temporal lobe epilepsy Epilepsy Behav 2011 21 88 90 10.1016/j.yebeh.2011.01.007 21317045 PMC13198858

[B9] Kauffman MA Consalvo D Moron DG Lereis VP Kochen S ApoE epsilon4 genotype and the age at onset of temporal lobe epilepsy: a case-control study and meta-analysis Epilepsy Res 2010 90 234 239 10.1016/j.eplepsyres.2010.05.007 20554432

[B10] Kauffman MA Pereira-de-Silva N Consalvo D Kochen S ApoE epsilon4 is not associated with posictal confusion in patients with mesial temporal lobe epilepsy with hippocampal sclerosis Epilepsy Res 2009 85 311 313 10.1016/j.eplepsyres.2009.03.012 19375284

[B11] Chapin JS Busch RM Janigro D Dougherty M Tilelli CQ Lineweaver TT Naugle RI Diaz-Arrastia R Najm IM *APOE *epsilon4 is associated with postictal confusion in patients with medically refractory temporal lobe epilepsy Epilepsy Res 2008 81 220 224 10.1016/j.eplepsyres.2008.05.003 18672349

[B12] Busch RM Lineweaver TT Naugle RI Kim KH Gong Y Tilelli CQ Prayson RA Bingaman W Najm IM Diaz-Arrastia R ApoE-epsilon4 is associated with reduced memory in long-standing intractable temporal lobe epilepsy Neurology 2007 68 409 414 10.1212/01.wnl.0000253021.60887.db 17283313

[B13] Kumar A Tripathi M Pandey RM Ramakrishnan L Srinivas M Luthra K Apolipoprotein E in temporal lobe epilepsy: a case-control study Dis Markers 2006 22 335 342 17264404 10.1155/2006/951632 PMC3850594

[B14] Yeni SN Ozkara C Buyru N Baykara O Hanoğlu L Karaağac N Ozyurt E Uzan M Association between APOE polymorphisms and mesial temporal lobe epilepsy with hippocampal sclerosis Eur J Neurol 2005 12 103 107 10.1111/j.1468-1331.2004.00956.x 15679697

[B15] Cavalleri GL Lynch JM Depondt C Burley MW Wood NW Sisodiya SM Goldstein DB Failure to replicate previously reported genetic associations with sporadic temporal lobe epilepsy: where to from here? Brain 2005 128 1832 1840 10.1093/brain/awh524 15888540

[B16] Gambardella A Aguglia U Chifari R Labate A Manna I Serra P Romeo N Sibilia G Lepiane E Russa AL Ventura P Cittadella R Sasanelli F Colosimo E Leggio U Zappia M Quattrone A ApoE epsilon4 allele and disease duration affect verbal learning in mild temporal lobe epilepsy Epilepsia 2005 46 110 117 10.1111/j.0013-9580.2005.15804.x 15660776

[B17] Briellmann RS Torn-Broers Y Busuttil BE Major BJ Kalnins RM Olsen M Jackson GD Frauman AG Berkovic SF *APOE *epsilon4 genotype is associated with an earlier onset of chronic temporal lobe epilepsy Neurology 2000 55 435 437 10932283 10.1212/wnl.55.3.435

[B18] Gambardella A Aguglia U Cittadella R Romeo N Sibilia G LePiane E Messina D Manna I Oliveri RL Zappia M Quattrone A Apolipoprotein E polymorphisms and the risk of nonlesional temporal lobe epilepsy Epilepsia 1999 40 1804 1807 10.1111/j.1528-1157.1999.tb01602.x 10612348

[B19] Blümcke I Brockhaus A Scheiwe C Rollbrocker B Wolf HK Elger CE Wiestler OD The apolipoprotein E epsilon 4 allele is not associated with early onset temporal lobe epilepsy Neuroreport 1997 8 1235 1237 10.1097/00001756-199703240-00035 9175120

[B20] Salzmann A Perroud N Crespel A Lambercy C Malafosse A Candidate genes for temporal lobe epilepsy: a replication study Neurol Sci 2008 29 397 403 10.1007/s10072-008-1060-9 19066720

[B21] Cramer PE Cirrito JR Wesson DW Lee CY Karlo JC Zinn AE Casali BT Restivo JL Goebel WD James MJ Brunden KR Wilson DA Landreth GE ApoE-directed therapeutics rapidly clear β-amyloid and reverse deficits in AD mouse models Science 2012 in press 10.1126/science.1217697 PMC3651582 22323736

[B22] Singh K Chaturvedi R Asim M Barry DP Lewis ND Vitek MP Wilson KT The apolipoprotein E-mimetic peptide COG112 inhibits the inflammatory response to Citrobacter rodentium in colonic epithelial cells by preventing NF-kappaB activation J Biol Chem 2008 283 16752 61 10.1074/jbc.M710530200 18417477 PMC2423260

[B23] Tukhovskaya EA Yukin AY Khokhlova ON Murashev AN Vitek MP COG1410, a novel apolipoprotein-E mimetic, improves functional and morphological recovery in a rat model of focal brain ischemia J Neurosci Res 2009 87 677 82 18803296 10.1002/jnr.21874 PMC2752425

[B24] Hoane MR Kaufman N Vitek MP McKenna SE COG1410 improves cognitive performance and reduces cortical neuronal loss in the traumatically injured brain J Neurotrauma 2009 26 121 9 19119914 10.1089/neu.2008.0565 PMC2749004

[B25] Sarantseva S Timoshenko S Bolshakova O Karaseva E Rodin D Schwarzman AL Vitek MP Apolipoprotein E-mimetics inhibit neurodegeneration and restore cognitive functions in a transgenic Drosophila model of Alzheimer's disease PLoS One 2009 4 12 e8191 PubMed PMID: 19997607; PubMed Central PMCID: PMC2782140 10.1371/journal.pone.0008191 19997607 PMC2782140

[B26] Li FQ Fowler KA Neil JE Colton CA Vitek MP An apolipoprotein E-mimetic stimulates axonal regeneration and remyelination after peripheral nerve injury J Pharmacol Exp Ther 2010 334 106 15 10.1124/jpet.110.167882 PMC2912037 20406857

[B27] Kaufman NA Beare JE Tan AA Vitek MP McKenna SE Hoane MR COG1410, an apolipoprotein E-based peptide, improves cognitive performance and reduces cortical loss following moderate fluid percussion injury in the rat Behav Brain Res 2010 214 395 401 20600347 10.1016/j.bbr.2010.06.017 PMC2936242

[B28] Laskowitz DT Song P Wang H Mace B Sullivan PM Vitek MP Dawson HN Traumatic brain injury exacerbates neurodegenerative pathology: improvement with an apolipoprotein E-based therapeutic J Neurotrauma 2010 27 1983 95 20812776 10.1089/neu.2010.1396

[B29] Christensen DJ Ohkubo N Oddo J Van Kanegan MJ Neil J Li F Colton CA Vitek MP Apolipoprotein E and peptide mimetics modulate inflammation by binding the SET protein and activating protein phosphatase 2A J Immunol 2011 186 2535 42 21289314 10.4049/jimmunol.1002847

[B30] Laskowitz DT Lei B Dawson HN Wang H Bellows ST Christensen DJ Vitek MP James ML The apoE-mimetic Peptide, COG1410, Improves Functional Recovery in a Murine Model of Intracerebral Hemorrhage Neurocrit Care 2012 16 316 26 21989844 10.1007/s12028-011-9641-5

[B31] Vitek MP Christensen DJ Wilcock D Davis J Van Nostrand WE Li FQ Colton CA APOE-Mimetic Peptides Reduce Behavioral Deficits, Plaques and Tangles in Alzheimer's Disease Transgenics Neurodegener Dis 2012 in press 10.1159/000334914 PMC3363346 22326991

